# On-Line Temperature Estimation for Noisy Thermal Sensors Using a Smoothing Filter-Based Kalman Predictor

**DOI:** 10.3390/s18020433

**Published:** 2018-02-02

**Authors:** Xin Li, Xingtao Ou, Zhi Li, Henglu Wei, Wei Zhou, Zhemin Duan

**Affiliations:** School of Electronics and Information, Northwestern Polytechnical University, Xi’an 710072, China; ouxingtao@mail.nwpu.edu.cn (X.O.); zhili@mail.nwpu.edu.cn (Z.L.); hengluw@mail.nwpu.edu.cn (H.W.); zhouwei@nwpu.edu.cn (W.Z.); zhemind@nwpu.edu.cn (Z.D.)

**Keywords:** dynamic thermal management (DTM), thermal sensor, noise, Kalman prediction, synergistic calibration, temperature estimation

## Abstract

Dynamic thermal management (DTM) mechanisms utilize embedded thermal sensors to collect fine-grained temperature information for monitoring the real-time thermal behavior of multi-core processors. However, embedded thermal sensors are very susceptible to a variety of sources of noise, including environmental uncertainty and process variation. This causes the discrepancies between actual temperatures and those observed by on-chip thermal sensors, which seriously affect the efficiency of DTM. In this paper, a smoothing filter-based Kalman prediction technique is proposed to accurately estimate the temperatures from noisy sensor readings. For the multi-sensor estimation scenario, the spatial correlations among different sensor locations are exploited. On this basis, a multi-sensor synergistic calibration algorithm (known as MSSCA) is proposed to improve the simultaneous prediction accuracy of multiple sensors. Moreover, an infrared imaging-based temperature measurement technique is also proposed to capture the thermal traces of an advanced micro devices (AMD) quad-core processor in real time. The acquired real temperature data are used to evaluate our prediction performance. Simulation shows that the proposed synergistic calibration scheme can reduce the root-mean-square error (RMSE) by 1.2 ${}^{\circ }$C and increase the signal-to-noise ratio (SNR) by 15.8 dB (with a very small average runtime overhead) compared with assuming the thermal sensor readings to be ideal. Additionally, the average false alarm rate (FAR) of the corrected sensor temperature readings can be reduced by 28.6%. These results clearly demonstrate that if our approach is used to perform temperature estimation, the response mechanisms of DTM can be triggered to adjust the voltages, frequencies, and cooling fan speeds at more appropriate times.

## 1. Introduction

The field of integrated circuit technology is entering the nanometer era. However, excessively increased power density leads to high chip temperature, which can result in thermal runaway. Elevated die temperature adversely affects the performance of multi-core processor systems, causing shortened lifetimes, increased cooling costs, and reduced reliability and device speed [1]. Therefore, reliable and effective thermal monitoring mechanisms are crucial to overcome this challenge. Dynamic thermal management (DTM) is often employed to continuously track the thermal behavior of processors during runtime [2]. Typically, on-die thermal sensors are widely deployed in modern multi-core processors to assist DTM [3]. According to the fine-grained temperature information collected by embedded thermal sensors, DTM techniques maintain the processor’s temperature within a preset range by reasonably assigning workload scheduling, and adjusting the voltages, frequencies, and cooling fan speeds appropriately [4,5]. In addition, in order to mitigate thermal emergencies on multi-core chips, only a fraction of cores can be simultaneously powered in the full performance mode, while other cores (i.e., dark cores) need to be power gated. In this so-called dark silicon problem [6,7,8,9] is important to ensure thermal-safe operation for modern chips, i.e., where the peak temperature does not exceed the safe-operating temperature, otherwise the response mechanisms of DTM are triggered.

**Figure 1:**
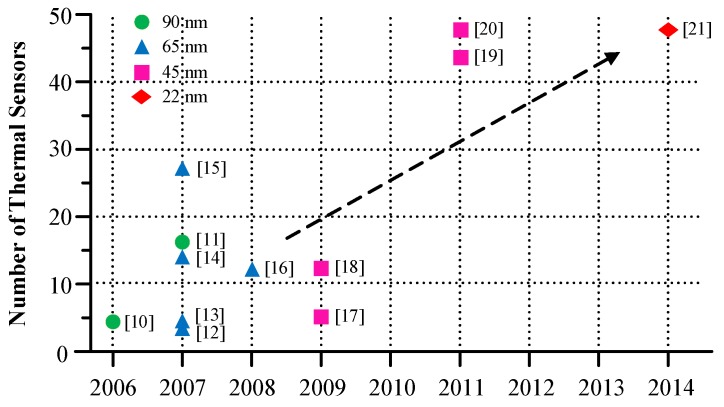
Trends in the number of embedded thermal sensors in VLSI systems.

The number of on-die thermal sensors keeps growing in very large scale integration (VLSI) systems to enable the DTM of chip functionalities [10,11,12,13,14,15,16,17,18,19,20,21], as shown in Figure 1. The accuracy of on-chip sensor readings has a great influence on the effectiveness and reliability of DTM. However, embedded thermal sensors are inevitably accompanied by noise, including process variation, supply voltage fluctuations, and cross-coupling etc, which cause the observed temperature readings to deviate from the actual values. In the worst case, the temperature reading error of un-calibrated thermal sensors used in IBM25PPC750L processors (International Business Machines Corporation (IBM), Armonk, New York, United States of America) can be up to 34 ${}^{\circ }$C (at an actual temperature of 95 ${}^{\circ }$C) [22]. Therefore, blindly trusting the thermal sensors to be ideal can lead DTM strategies to make inaccurate decisions that result in false alarms or unnecessary responses.

Thermal monitoring and management in VLSI systems have been widely researched in recent years [23,24,25]. Nowroz et al. [26] utilized frequency-domain signal representations to devise both static and runtime thermal monitoring approaches. Unfortunately, this work does not consider the effect of inaccurate and noisy sensors. Reda et al. [27] proposed a new direction to simultaneously identify the thermal models and the fine-grain power consumption of a chip from just the measurements of the thermal sensors and the total power consumption. Although they verified the accuracy of this method and demonstrated its resilience to sensor noise, the problem of noise reduction for sensor measurements was not addressed. Effective temperature calibration can compensate for inaccuracies in temperature measurement, and help to improve thermal sensing accuracy. As a result, how to solve the problem of estimating temperatures for on-chip thermal sensors corrupted by noise is a major challenge.

A number of studies have taken into account the noise issue associated with sensor readings, such as the statistical methodology [28] and the multi-sensor collaborative calibration algorithm (MSCCA) [29]. However, these techniques lack the ability for real-time prediction which is required for proactive DTM techniques [30]. In [31,32], the authors proposed a scheme to make online temperature measurements significantly more accurate. They constructed an offline thermal equivalent resistor–capacitor (RC) model and reduced its complexity by a projection-based model order reduction method. This model can be used to convert the power dissipation to temperature in the prediction step of the Kalman filter. However, the derivation of such an RC model is not trivial due to the complexity of silicon materials. Unlike the above approach, we apply the polynomial fitting technique to convert the oscillation frequency of noisy sensors to temperature data and use the smoothing filter to obtain the prediction information. These two sources of temperature information are then combined in the Kalman filter to generate reliable temperature estimations. This direct method reduces the calibration cost because it eliminates the requirement for estimating the power consumption per functional unit. Specifically, the contributions of this work are as follows:

- The noise characteristics of on-chip thermal sensors based on the ring oscillator structure are systematically analyzed. On this basis, the polynomial fitting technique is used to establish the non-linear relationship between sensor temperature and oscillation frequency, which can improve the measurement accuracy.
- To tackle the challenge in temperature estimation of noisy thermal sensors, a smoothing filter-based Kalman prediction technique is proposed to correct the temperatures of on-die sensors in real-time.
- For the multi-sensor estimation scenario, the spatial correlations among different sensor locations are exploited. On this basis, a multi-sensor synergistic calibration algorithm (called MSSCA) is proposed to improve the simultaneous prediction accuracy of multiple sensors.
- Relative to the previous works relied on computer-based thermal simulation scheme, an infrared imaging-based temperature measurement technique is proposed to provide the accurate thermal characterizations of an AMD quad-core processor operating on different benchmarks. The captured real temperature data are used to evaluate our prediction approach.

The remainder of this paper is organized as follows: Section 2 provides the necessary motivation of this work, and presents the analysis of noisy sensor behavior. Section 3 presents the on-line temperature estimation technique using a smoothing filter-based Kalman predictor, and details the proposed multi-sensor synergistic calibration algorithm (MSSCA) that can improve the simultaneous prediction accuracy of multiple sensors. Section 4 describes the infrared temperature measurement setup required for capturing the thermal traces of real processors. The performance of our synergistic calibration scheme is validated in Section 5. Finally, we summarize the main conclusions of this work in Section 6.

## 2. Analysis of Noisy Sensor Behavior

**Figure 2:**
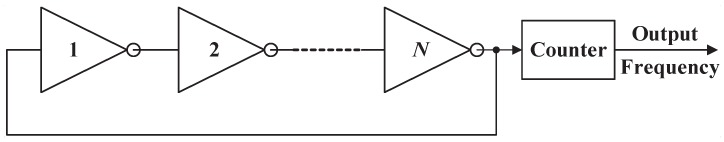
Structure of a typical ring oscillator.

Due to the unpredictable behavior of the chip’s thermal profile, constantly monitoring the processor’s temperature using embedded thermal sensors is critical to ensuring long-term reliability of integrated circuit systems. A classical implementation of on-die thermal sensors is the ring oscillator, which has been known for nearly 30 years [33]. The structure of a typical ring oscillator mainly contains *N* (an odd number) stages of inverters and a counter, as depicted in Figure 2. Note that the output frequency of ring oscillator in Figure 2 represents the oscillation frequency.

The output frequency depends on the total time-delay of inverters, which is given by the following equation:

(1) $$f=\frac{1}{N(t_{HL}+t_{LH)}}$$

where $t_{HL}\left(t_{LH}\right)$ denotes the time-delay with a single inverter switching from the high (low) voltage to the low (high) voltage. The expression for $t_{HL}$ can be described as:

(2) $$t_{HL}=\frac{2C}{\mu_{n}C_{ox}{\left(W/L\right)}_{n}\left(V_{DD}-V_{t}\right)}\times \left[\frac{V_{t}}{V_{DD}-V_{t}}+\frac{1}{2}\ln\left(\frac{3V_{DD}-4V_{t}}{V_{DD}}\right)\right]$$

where *C* and $C_{ox}$ are the effective load capacitance and the oxide capacitance per unit gate area, $V_{DD}$ and $V_{t}$ are the supply voltage and the threshold voltage, and ${\left(W/L\right)}_{n}$ and $\mu_{n}$ are the width/length ratio and the electron mobility of n-metal-oxide-semiconductor (NMOS), respectively. Note that the expression for $t_{LH}$ is identical to Equation (2) except that ${\left(W/L\right)}_{n}$ and $\mu_{n}$ need to be replaced with the corresponding parameters of p-metal-oxide-semiconductor (PMOS), i.e., ${\left(W/L\right)}_{p}$ and $\mu_{p}$, respectively. According to Equations (1) and (2), the output frequency is easily affected by temperature since both $V_{t}$ and $\mu_{n}\left(\mu_{p}\right)$ are sensitive to temperature. To describe the temperature effects more accurately, the following empirical equations can be used [34]:

(3) $$V_{t}\left(T\right)=V_{t}\left(T_{0}\right)-0.002\left(T-T_{0}\right)$$

(4) $$\mu_{n/p}\left(T\right)=\mu_{n/p}\left(T_{0}\right){\left(T/T_{0}\right)}^{-1.5}$$

where $T_{0}$ is the reference temperature. As we can see from Equations (3) and (4), $V_{t}$ drops by 2 mV when temperature increases by 1 ${}^{\circ }$C, and $\mu_{n}\left(\mu_{p}\right)$ also decreases with a more complex relationship when temperature rises. Because $\mu_{n}\left(\mu_{p}\right)$ dominates in the influence of time-delay, the overall effect appears a decrease of output frequency with temperature rise. Therefore, the output frequency observations can be used to measure the chip temperature.

However, due to environmental uncertainty and process variation, the accuracy of output frequency is highly susceptible to several factors, such as variations in process parameters and fluctuations in supply voltage and ambient temperature. Generally, these noise sources can be divided into two main categories, i.e., dynamic noise and static noise. Dynamic noise represents the variation in the accuracy of specific sensors over time, which is caused by fluctuations in supply voltage and ambient temperature. Static noise means variations in the circuit parameters, including load capacitance, oxide capacitance, length/width ratio, etc. To describe the statistical characteristics of output frequency observations under the influence of noise, the following Monte Carlo (MC) simulation is performed using Equations (1)–(4) with 100,000 samples for each different temperature, ranging from 30 ${}^{\circ }$C to 70 ${}^{\circ }$C with an increment of 10 ${}^{\circ }$C. All the random variables are assumed to be the normal distribution with mean values and standard deviations specified in Table 1.

**Figure 3:**
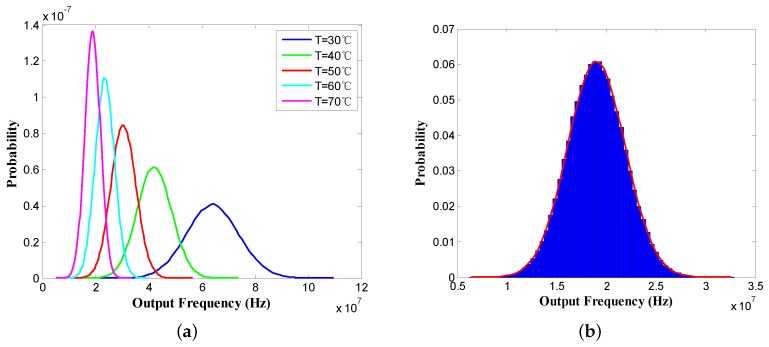
Results of the Monte Carlo (MC) simulation. (**a**) Probability density distribution of output frequencies under different temperatures; (**b**) Statistical histogram of output frequency distribution at the temperature of 70 ${}^{\circ }$C.

The results of the MC simulation are given in Figure 3. Specifically, the probability density distribution of output frequencies under different temperatures is depicted in Figure 3a. Each curve in Figure 3a shows the potential distribution of output frequencies for a fixed sensor temperature. The integral of the probability density under each curve over the entire space is equal to one. From Figure 3a, it can be observed that these probability density distribution curves heavily overlap with each other, i.e., the same output frequency could be caused by multiple potential temperatures. Therefore, blindly trusting the thermal sensors to be ideal could lead to significant error. This clearly indicates that effective temperature estimation methods are very important for predicting the accurate temperatures from noisy sensor readings. Besides, the statistical histogram of output frequency distribution at the temperature of 70 ${}^{\circ }$C is shown in Figure 3b. The statistical histogram divides the entire range of values into a series of intervals, and then counts how many values fall into each interval. In Figure 3b, we divide the entire range of frequencies for the pink curve into 60 intervals on average. The height of each rectangle in Figure 3b indicates the sum of probabilities which fall into the corresponding interval.

## 3. Temperature Estimation for Noisy Thermal Sensors

**Figure 4:**
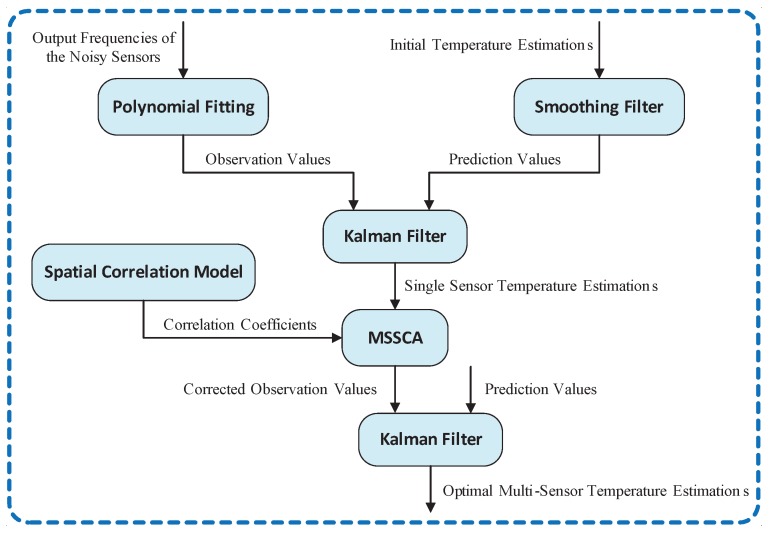
Flowchart of the proposed scheme for temperature estimation. MSSCA: multi-sensor synergistic calibration algorithm.

Based on the above analysis of noisy sensor behavior, an accurate on-line temperature estimation technique is proposed in this section, which can be divided into the following five steps. The flowchart of our proposed scheme is given in Figure 4.

**Figure 5:**
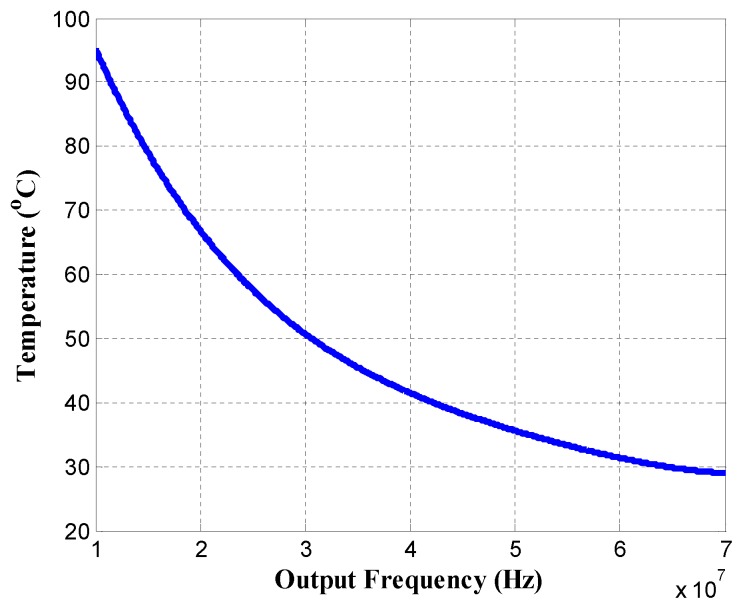
Result of the polynomial fitting.

- Step 1: Establish the non-linear relationship between sensor temperature and output frequency using the polynomial fitting technique, and then calculate the temperature observation values of noisy sensors.
   Based on Equations (1)–(4), we use the mean values of random variables specified in Table 1 to generate the observed data of output frequencies by varying the sensor temperature. The reference temperature is set to 25 ${}^{\circ }$C. The actual temperature data is acquired by our infrared temperature measurement setup (described later in Section 4). Using the observed data, the non-linear relationship between sensor temperature and output frequency can be established by the polynomial fitting. The fitting result is shown in Figure 5.
- Step 2: Establish the temperature prediction model using the smoothing filter, and calculate the temperature prediction values of noisy sensors.
- Step 3: Correct the temperatures of noisy sensors using the Kalman filter.
- Step 4: Establish the spatial correlation model, and update the temperature observation values using the multi-sensor synergistic calibration algorithm (MSSCA).
- Step 5: Reuse the Kalman filter to calculate the optimal multi-sensor temperature estimations.

### 3.1. Smoothing Filter-Based Kalman Prediction Technique

The Kalman filter [35], also known as linear quadratic estimation, is an efficient recursive filter that estimates the internal state of a linear dynamic system from a series of noisy measurements and is popular for its simple implementation and computational complexity. It has been widely used in numerous engineering applications. Recently, the methods based on the Kalman filter have shown the ability to track the temperature profile of a chip in real time. In order to apply the Kalman filter algorithm to predict the temperatures from noisy sensor observations, it is essential to introduce the state space model which is governed by the linear difference Equation (5). Considering the effect of noise and inaccuracy, the observation model is described as Equation (6).

(5) T(k+1)=BT(k)+w(k)

(6) S(k)=HT(k)+v(k)

Here, at time instant *k*, T(k) is the state vector representing the predictions, S(k) is the measurement vector representing the sensor readings, and w(k) and v(k) are the process noise vector and the measurement noise vector, respectively. The coefficient matrices B and H denote the state matrix and the output matrix, respectively.

According to Equations (5) and (6), the Kalman filter algorithm can be employed to estimate the process in a recursive manner, which consists of two distinct phases, namely predict and update. Using the state estimate from the previous time step, the predict phase can generate a priori temperature estimate and error covariance at the current time step. Recently, smoothing filters [36] have attracted significant attention since they work well for many denoising problems. One of the most common smoothing algorithms is the moving average (MA), which is often used to attempt to capture important trends in the observed data. Based on the characteristic that the chip temperature does not suddenly change within short temporal sampling interval, smoothing filters can be used to minimize the impacts of temperature fluctuations. Therefore, a simple moving average (SMA) shown in Equation (7) is designed to achieve a more accurate temperature prediction model. Consequently, the equations of predict phase can be updated as:

(7) $$\hat{T}\left(k|k-1\right)=B\left[\left(\sum_{t=k-L_{s}}^{t=k-1}\hat{T}\left(t|t\right)\right)/L_{s}\right]$$

(8) $$P\left(k|k-1\right)=BP\left(k-1|k-1\right)B^{T}+Q$$

where $L_{s}$ represents the length of the smoothing window, Q is the covariance matrix of the process noise, $\hat{T}(k|k-1)$ is the priori state estimate vector, and P(k|k−1), and P(k−1|k−1) denote the priori and posteriori error covariance matrix, respectively. The first prediction value is obtained by taking the average of initial temperature estimations in the smoothing window, and then the prediction value is dynamically modified by shifting the window forward. In our case, initial temperature estimations are set to noisy sensor readings. The effects of initial temperature estimations on the smoothing filter are less obvious. However, the length of the smoothing window will directly affect the prediction performance. We have experimentally determined that SMA works best when the length of the smoothing window is equal to 5.

In the update phase, the current priori prediction is combined with the current observation information to obtain an improved posteriori state estimate. The equations of update phase can be expressed as:

(9) $$K\left(k\right)=P\left(k|k-1\right)H^{T}{\left[HP\left(k|k-1\right)H^{T}+R\right]}^{-1}$$

(10) $$\hat{T}(k|k)=\hat{T}(k|k-1)+K(k)[S(k)-H\hat{T}(k|k-1)]$$

**Figure 6:**
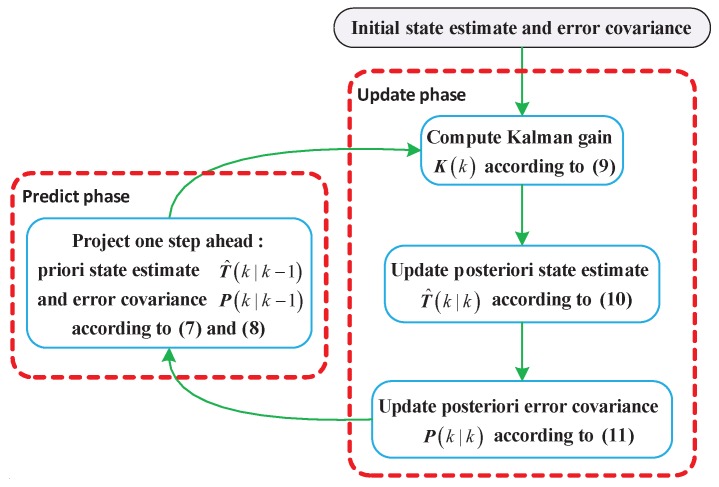
Framework of the Kalman filter algorithm.

(11) P(k|k)=[I−K(k)H]P(k|k−1)

where K(k) represents the Kalman gain matrix, and R denotes the covariance matrix of measurement noise. The framework of the Kalman filter algorithm is illustrated in Figure 6.

### 3.2. Multi-Sensor Synergistic Calibration Algorithm (MSSCA)

**Figure 7:**
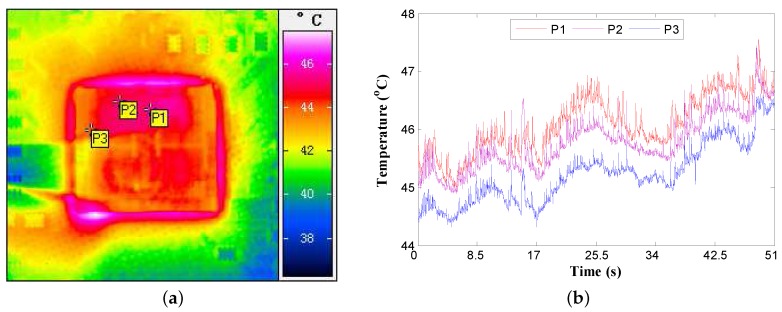
Distribution of sensor locations and the corresponding temperature variations. (**a**) Distribution of sensor locations; (**b**) Thermal traces of different sensor locations.

Current VLSI chips deploy multiple sensors to continuously monitor the thermal state of different overheating positions. One important observation is that temperature variations at different sensor locations on the chip are correlated. Typically, sensors close to each other physically are likely to have stronger spatial correlation than sensors far apart [30,37]. Such correlations are caused by similar power behaviors. This phenomenon can be observed by our infrared temperature measurement setup (described later in Section 4). To illustrate the spatial correlation of temperature variations, the temperatures of three different sensor locations are captured, as shown in Figure 7. In particular, the distribution of sensor locations is given in Figure 7a, and the corresponding temperature variations are shown in Figure 7b. The thermal traces confirm that nearby sensors have similar characteristics compared with sensors far apart. Moreover, the variability in process parameters (such as channel width, length, and oxide thickness) can also be correlated, which results in the correlated noisy behavior of sensors. Such correlation models can be established by the statistical static timing analysis (SSTA). In our methodology, the correlation information of fabrication randomness at different sensor locations is considered as well. As compared with treating each sensor independently, the spatial correlation can be used to correct the sensor observations so as to further improve the accuracy of temperature estimation. Therefore, exploiting the spatial correlation is necessary.

**Figure 8:**
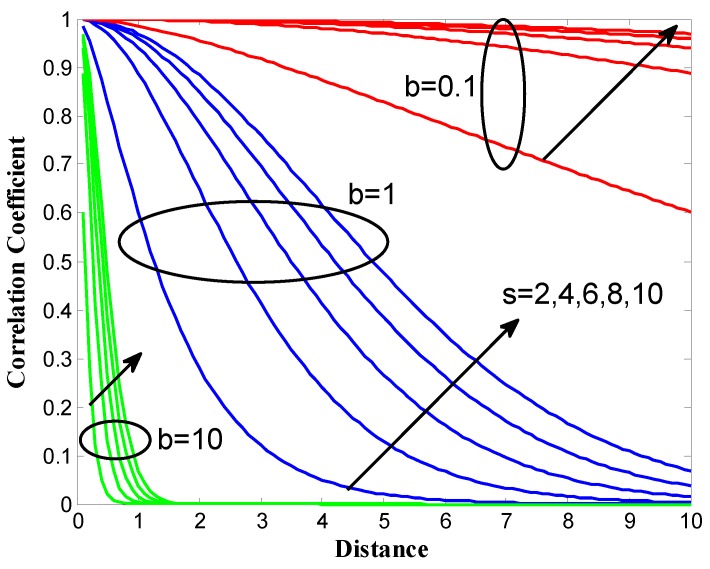
Spatial correlation functions generated from Equation (12).

There are some studies aimed at the modeling of the spatial correlation [38,39,40]. In [40], the authors applied mathematical theories from random fields and convex analysis to develop robust techniques to extract a valid spatial correlation function from measurement data, and they have experimentally confirmed that the resulting correlation function is the closest ones to the underlying model even if the data are affected by unavoidable random noise. Therefore, the spatial correlation function proposed in [40] is adopted in our methodology. The spatial correlation coefficient (ρ) between any two different sensor positions can be described as:

(12) $$\rho_{i,j}=2{\left(\frac{bv_{i,j}}{2}\right)}^{s-1}\kappa_{s-1}\left(bv_{i,j}\right)\Gamma {\left(s-1\right)}^{-1}$$

where $\kappa_{s-1}\left(\cdot \right)$ represents the modified Bessel function of the second kind of order (s−1), Γ(·) is the gamma function, and $v_{i,j}$ denotes the Euclidean distance between two sensor locations on the chip which is expressed as follows:

(13) $$v_{i,j}=\sqrt{{\left(x_{i}-x_{j}\right)}^{2}+{\left(y_{i}-y_{j}\right)}^{2}}$$

where $\left(x_{i},y_{i}\right)$ and $\left(x_{j},y_{j}\right)$ denote the coordinates of any two sensors. The shape of the spatial correlation function is regulated by the two real parameters *b* and *s*. To facilitate for our spatial correlation modeling, a rich set of correlation functions can be obtained by varying *b* and *s*, as shown in Figure 8. In our case, *b* and *s* are set to 1 and 8, respectively.

Based on the aforementioned spatial correlation model, the multi-sensor synergistic calibration algorithm (MSSCA) is devised to correct the sensor measurements using the correlations. The goal of the MSSCA is to further improve the simultaneous prediction accuracy of multiple sensors. For one arbitrary sensor (denoted as *m*) of all the *M* sensors in the monitored region, the MSSCA can be presented in four steps as follows:

1. Compute the correlation coefficients $\rho_{m,i}$ ($0\leq \rho_{m,i}\leq 1$, for 1≤i≤M and i≠M) of sensor *m* with all the other sensors, and pick out the largest one ($\rho_{m,n}$), i.e., sensor *m* has the strongest correlation with sensor *n*.
2. Set the correlation threshold λ. If $\rho_{m,n}\leq \lambda$, the temperature measurement of sensor *m* will not be updated, i.e., ${Ŝ}_{m}\left(k\right)=S_{m}\left(k\right)$; otherwise, the temperature observation of sensor *m* can be corrected as:
  (14) $${Ŝ}_{m}\left(k\right)=\left\{\begin{array}{c} S_{m}\left(k\right)+\frac{\rho_{m,n}\left|S_{n}\left(k\right)-{\hat{T}}_{n}\left(k|k\right)\right|}{2},\;S_{m}\left(k\right)<{\hat{T}}_{m}\left(k|k\right)\;\mathrm{and}\;S_{n}\left(k\right)<{\hat{T}}_{n}\left(k|k\right)\\ S_{m}\left(k\right)-\frac{\rho_{m,n}\left|S_{n}\left(k\right)-{\hat{T}}_{n}\left(k|k\right)\right|}{2},\;S_{m}\left(k\right)>{\hat{T}}_{m}\left(k|k\right)\;\mathrm{and}\;S_{n}\left(k\right)>{\hat{T}}_{n}\left(k|k\right) \end{array}\right.$$
3. Perform steps 1–2 in the residual sensors until the temperature observation of each sensor has been completed in the calibration, and then update the corresponding measurement vector to $Ŝ\left(k\right)=\{{Ŝ}_{1}\left(k\right),{Ŝ}_{2}\left(k\right),\ldots ,{Ŝ}_{m}\left(k\right)\}$.
4. Calculate the optimal temperature predictions using the following equation:
  (15) $$\hat{T}(k|k)=\hat{T}(k|k-1)+K(k)[Ŝ(k)-H\hat{T}(k|k-1)]$$

The pseudo code of the MSSCA is shown in Algorithm 1. Note that the correlation coefficients among all the available sensors comprise the coefficient matrix (ρ) of dimension [M×M], and ρ is a symmetric matrix in which the elements on the diagonal are all equal to one, as shown in Equation (16).

**Algorithm 1** Multi-Sensor Synergistic Calibration Algorithm (MSSCA)1.**Initialize:**
$\hat{T}\left(k|k\right)=S\left(k\right),k=1,2,\ldots ,L_{s}$2.Compute the coefficient matrix ρ according to Equation (12)3.Remove the autocorrelation by ρ=ρ−I, and store ρ in memory4.***maxc***(1:M)=0, and ***maxl***(1:M)=05.**for**
i=1 to *M*
**do**6. **for**
j=1 to *M*
**do**7.  **if**
maxc(i)<ρ(i,j)
**then**8.   maxc(i)=ρ(i,j), and maxc(i)=j9.  **end if**10. **end for**11.**end for**12.**for**
$k=(L_{s}+1)$ to *K*
**do**13. smoothsum=014. **for**
$t=(k-L_{s})$ to (k−1)
**do**15.  $smoothsum=smoothsum+\hat{T}(t|t)$16. **end for**17. $\hat{T}\left(k|k-1\right)=B\left(smoothsum/L_{s}\right)$18. $P\left(k|k-1\right)=BP\left(k-1|k-1\right)B^{T}+Q$19. $K\left(k\right)=P\left(k|k-1\right)H^{T}{\left[HP\left(k|k-1\right)H^{T}+R\right]}^{-1}$20. $\hat{T}(k|k)=\hat{T}(k|k-1)+K(k)[S(k)-H\hat{T}(k|k-1)]$21. P(k|k)=[I−K(k)H]P(k|k−1)22. **for**
i=1 to *M*
**do**23.  **if**
maxc(i)<λ
**then**
${Ŝ}_{i}\left(k\right)=S_{i}\left(k\right)$24.  **else if**
$S_{i}\left(k\right)<{\hat{T}}_{i}\left(k|k\right)\;\&\&\;S_{\mathrm{maxl}(i)}\left(k\right)<{\hat{T}}_{\mathrm{maxl}(i)}\left(k|k\right)$
**then**25.   ${Ŝ}_{i}\left(k\right)=S_{i}\left(k\right)+\frac{\rho \left(i,\mathrm{maxl}\left(i\right)\right)|S_{\mathrm{maxl}(i)}\left(k\right)-{\hat{T}}_{\mathrm{maxl}(i)}\left(k|k\right)|}{2}$26.  **else if**
$S_{i}\left(k\right)>\hat{T}\left(k|k\right)\;\&\&\;S_{\mathrm{maxl}(i)}\left(k\right)>{\hat{T}}_{\mathrm{maxl}(i)}\left(k|k\right)$
**then**27.   ${Ŝ}_{i}\left(k\right)=S_{i}\left(k\right)-\frac{\rho \left(i,\mathrm{maxl}\left(i\right)\right)|S_{\mathrm{maxl}(i)}\left(k\right)-{\hat{T}}_{\mathrm{maxl}(i)}\left(k|k\right)|}{2}$28.  **else**
${Ŝ}_{i}\left(k\right)=S_{i}\left(k\right)$29.  **end if**30. **end for**31. $\hat{T}(k|k)=\hat{T}(k|k-1)+K(k)[Ŝ(k)-H\hat{T}(k|k-1)]$32.**end for**

(16) $$\rho =\left[\begin{array}{cccc} 1 & \rho_{1,2} & \cdots & \rho_{1,M}\\ \rho_{2,1} & 1 & \cdots & \rho_{2,M}\\ \vdots & \vdots & \ddots & \vdots \\ \rho_{M,1} & \rho_{M,2} & \cdots & 1 \end{array}\right]$$

To remove the autocorrelation, the coefficient matrix can be built as ρ=ρ−I, where I denotes the identity matrix. Considering the correlation coefficient only depends on the distance that is not changed because the placement of sensors was fixed at design time, the correlation coefficients only need to be calculated when the MSSCA is first implemented, and then the upper triangular matrix of ρ is stored in memory.

## 4. Infrared Imaging-Based Temperature Measurement Technique

The inputs to our temperature estimation technique are the thermal traces at a set of discrete sensor positions. These inputs could be generated from either a computer-based thermal simulator or an infrared imaging-based thermal measurement infrastructure. The previous related studies on thermal tracking mainly rely on computer-based simulations. To obtain the thermal traces, these simulations utilize the workload power traces from an architectural-level simulator (e.g., Wattch [41]) together with the floor-plan of processor as inputs to a temperature simulator (e.g., Hotspot [42]). In this section, an infrared imaging-based temperature measurement setup is developed to obtain the accurate thermal characterizations of an AMD quad-core processor operating on different benchmark workloads. Recent studies on thermal measuring have confirmed the value of the complementary information that infrared thermography provided [43,44,45].

**Figure 9:**
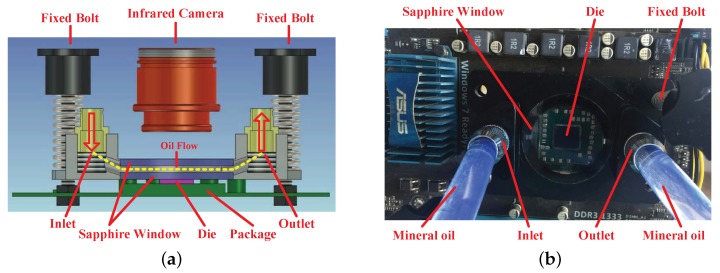
Proposed infrared temperature measuring equipment. (**a**) Oil-based cooling system; (**b**) Infrared transparent heat sink.

**Figure 10:**
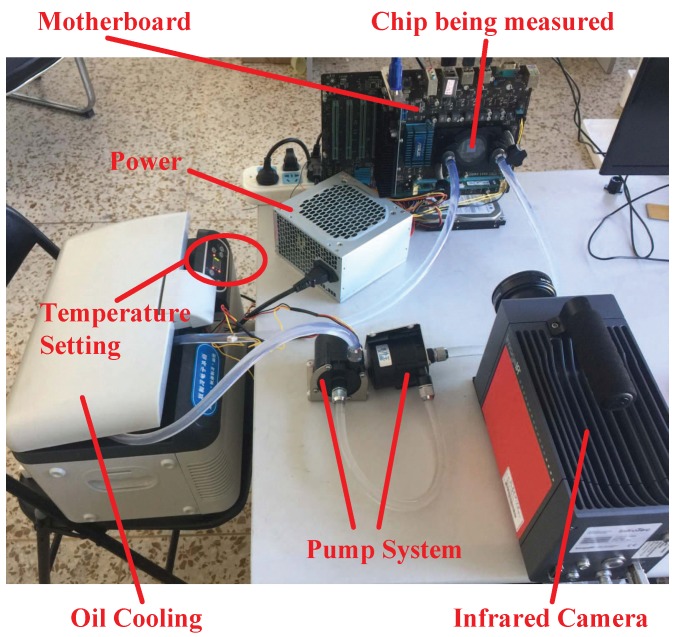
Image of our experimental setup.

**Figure 11:**
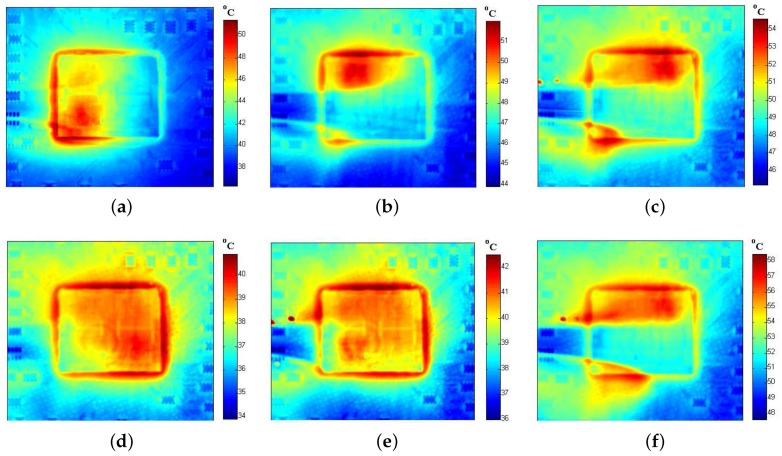
Examples of thermal traces of different benchmarks on a quad-core AMD Athlon II X4 610e processor. (**a**) sjeng; (**b**) h264ref; (**c**) lbm; (**d**) leslie3d; (**e**) sphinx3; (**f**) dealII.

To track the thermal behavior of processor in real-time, an oil-based cooling system is designed to replace the infrared opaque metal heat sink. Once the original metal heat sink is removed, we need an infrared transparent heat sink to dissipate the generated heat adequately. To keep the chip working within a safe temperature range, a distinctive heat sink is devised that contains two layered sapphire windows (with an around 4-mm thickness for each). The proposed infrared temperature measuring equipment is depicted in Figure 9. As compensation for the conventional thermal interface material (TIM), the sapphire window on the top of the die is used to improve lateral heat spreading and increase the thermal capacitance. Due to the relatively high thermal conductivity, large specific heat capacity, and good transparency in the infrared range, mineral oil is a suitable choice for a coolant [44,45]. The mineral oil (Sigma M3156 (Sigma-Aldrich Corporation, St. Louis, Missouri, United States of America)) is persistently pushed through the inlet by an external direct current (DC) pump that circulates between the two layers of sapphire window to transfer the heat. In order to keep the flow laminar, the clearance of two sapphire windows is restricted to 1 mm. The oil temperature is monitored using a thermostat. The detailed thermal traces of the SPEC CPU 2006 (Standard Performance Evaluation Corporation (SPEC), Gainesville, Virginia, United States of America) benchmark workloads [46] are captured using a mid-wave infrared camera (InfraTec ImageIR^®^ 8300 (InfraTec GmbH Infrarotsensorik und Messtechnik, Dresden, Germany)). Because the lightly doped and undoped silicon are partially transparent at the mid-wave infrared range, the chip temperature can be measured through our tailored infrared transparent heat sink. The chip being tested is a 45-nm AMD Athlon II X4 610e (Advanced Micro Devices, Inc. (AMD), Santa Clara, California, United States of America) quad-core processor [47] operating at 2.4 GHz. The image of our experimental setup is exhibited in Figure 10. To demonstrate the effectiveness of our infrared thermography technique, a few examples of thermal traces are shown in Figure 11.

## 5. Experimental Results

In this section, the performance of our temperature estimation approach is verified using the real thermal traces obtained by the above infrared temperature measurement setup. In what follows, we consider the case that three thermal sensors (denoted as P1, P2, and P3) are placed on the chip (see Figure 7a), and we try to calibrate their temperatures from the noisy sensor observations. It should be noted that the approach is the same for more than three sensors. The correlation coefficients among all three sensors are calculated according to Equation (12) as shown in Table 2, and the correlation threshold (λ) is set to 0.8. The random parameters of thermal sensors are assumed to be of normal distribution, and we set the mean values of these parameters to be the standard values used in the 180-nm fabrication process (see Table 1). The dynamic noise source is assumed to have a supply voltage ($V_{DD}$) fluctuation. Then, the MC simulation is performed based on Equations (1)–(4) to generate the noisy sensor readings that are used to test our temperature estimation. All the simulations are implemented by MATLAB code running on an Intel Core (Intel Corporation, Santa Clara, California, United States of America) 3.2 GHz computer with 16 GB synchronous dynamic random access memory (SDRAM).

**Figure 12:**
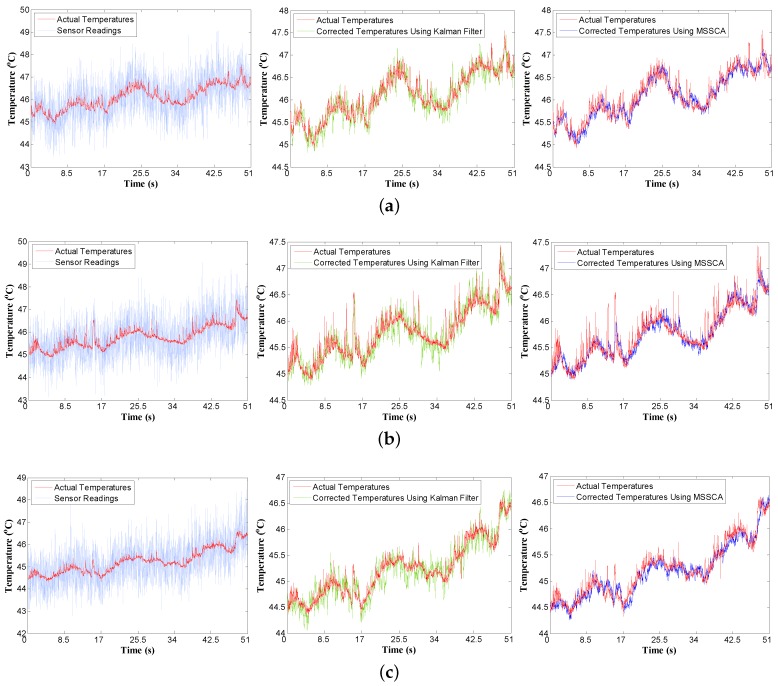
Comparison of temperature tracking (over 51 s) for three sensors in Figure 7a running the gamess benchmark. Noise standard deviation is 5%. (**a**) P1 sensor; (**b**) P2 sensor; (**c**) P3 sensor. MSSCA: multi-sensor synergistic calibration algorithm.

**Figure 13:**
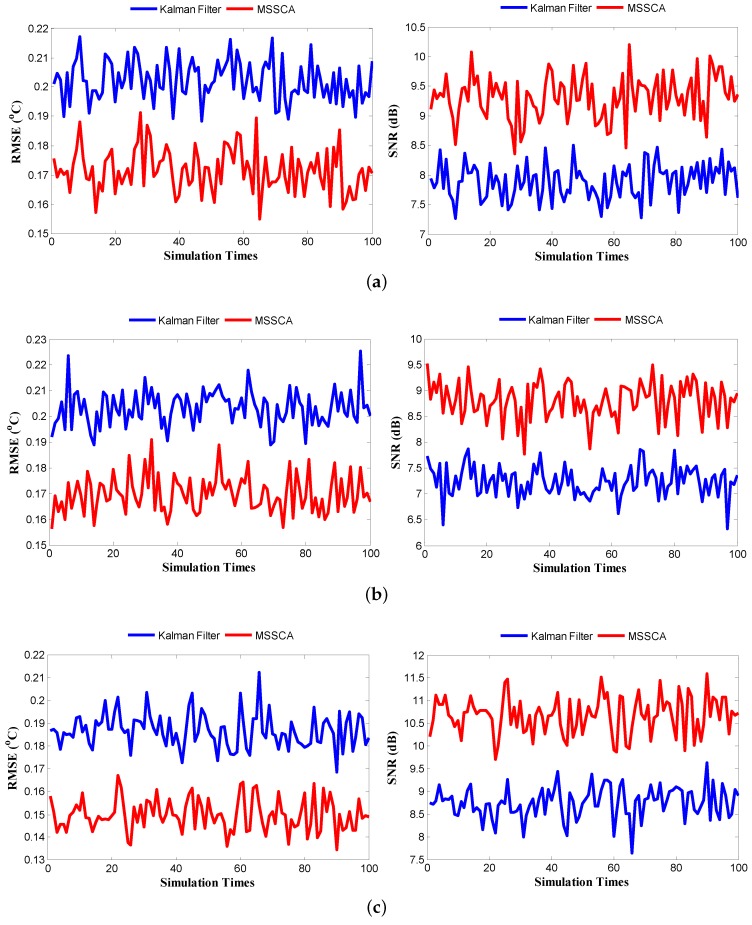
Root-mean-square error (RMSE) and signal-to-noise ratio (SNR) generated from the Kalman filter and the MSSCA with 100 time instances for three sensors in Figure 7a running the gamess benchmark. Noise standard deviation is 5%. (**a**) P1 sensor; (**b**) P2 sensor; (**c**) P3 sensor.

**Figure 14:**
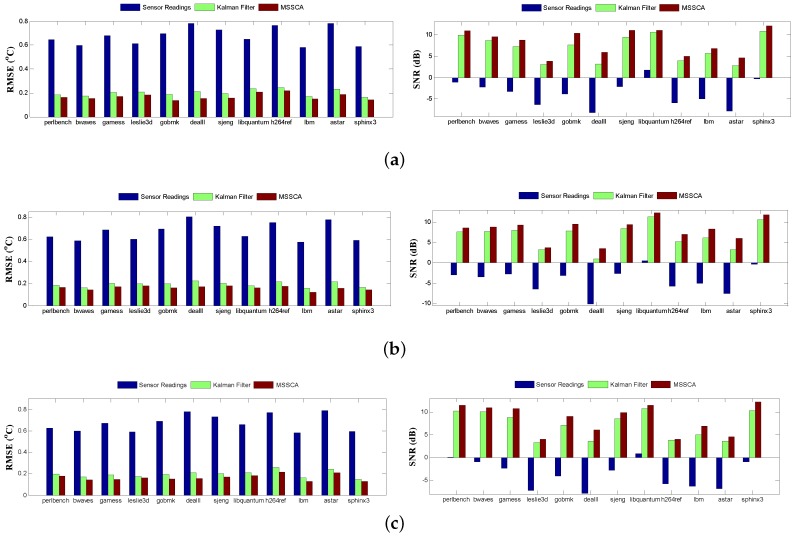
Comparison of prediction accuracy for different benchmarks. Noise standard deviation is 5%. (**a**) P1 sensor; (**b**) P2 sensor; (**c**) P3 sensor.

Figure 12 highlights the temperature tracking results of three sensors running the gamess benchmark. The standard deviation of the $V_{DD}$ is set to be 5% of its mean value. The results of other benchmarks are similar. There are four different color curves plotted in the figure for each sensor: actual temperatures, noisy sensor readings, Kalman filter-corrected temperatures, and MSSCA-corrected temperatures. The simulation lasts for 51 s, and contains 3000 sample points, i.e., the sampling interval is 17 ms. From the results, it can be observed that the predicted temperatures using the MSSCA are much closer to the actual temperatures than those using the Kalman filter. In addition, the comparison results of the root-mean-square error (RMSE) and the signal-to-noise ratio (SNR) generated from the Kalman filter and MSSCA are shown in Figure 13. Experiments are performed with 100 time instances. From the results, it can be seen that the prediction performance of the MSSCA is clearly superior to that of the Kalman filter, with a lower RMSE and a higher SNR under all three sensors. Furthermore, an intuitive comparison of the prediction accuracy for different benchmarks is given in Figure 14. In Figure 14b (see the dealII benchmark), the RMSE of MSSCA can be reduced by 0.6 ${}^{\circ }$C (from 0.8 ${}^{\circ }$C to 0.2 ${}^{\circ }$C) relative to the noisy sensor readings. In Figure 14a (see the gobmk benchmark), the SNR of MSSCA is increased by 14.1 dB (from −3.8 dB to 10.3 dB) compared with the original sensor readings.

The results of average prediction accuracy under different noise standard deviations are reported in Table 3. Comparing the results, it can be observed that the MSSCA still exhibits superior prediction performance even if the noise standard deviation increases. In the case of 10% noise standard deviation, MSSCA can obtain a 1.2 ${}^{\circ }$C reduction in RMSE and a 15.8 dB increment in SNR compared with assuming the sensor readings to be ideal. Compared with Kalman filter, the average prediction accuracy increments of the MSSCA are reported in Table 4. As seen in Table 4, our MSSCA can achieve a 17.9% reduction in RMSE and a 45.8% increment in SNR. Note that the results of improvement for three sensors are slightly different. This is because the trends in temperature variation and the characteristics of observation data for different sensors lead to different degrees of improvement for prediction performance.

**Figure 15:**
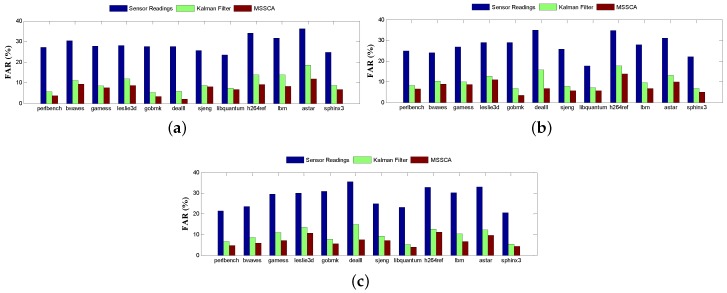
Comparison of the false alarm rate (FAR) for different benchmarks. Noise standard deviation is 5%. (**a**) P1 sensor; (**b**) P2 sensor; (**c**) P3 sensor.

Another potential impact on DTM is the false alarm rate (FAR) [48], which is derived from two emergencies, i.e., missed and fake. The former indicates that the actual temperatures have exceeded the threshold temperature, but the estimated temperatures are still below it, and vice versa for the latter. The FAR comparison for different benchmarks is depicted in Figure 15, and the average results under different noise standard deviations are summarized in Table 5. In our case, the temperature threshold is set to 95% of the maximum temperature for each benchmark, where DTM will be triggered to cut down the frequencies. As seen in Table 5, MSSCA can achieve a 28.6% reduction in FAR as compared to the noisy sensor observations. The results clearly demonstrate that if our MSSCA is used to perform the temperature estimation, the performance of DTM can be significantly improved. This is because DTM mechanisms (e.g., dynamic voltage and frequency scaling (DVFS)) can be triggered to adjust the voltages, frequencies, and fan speeds at more appropriate times.

**Figure 16:**
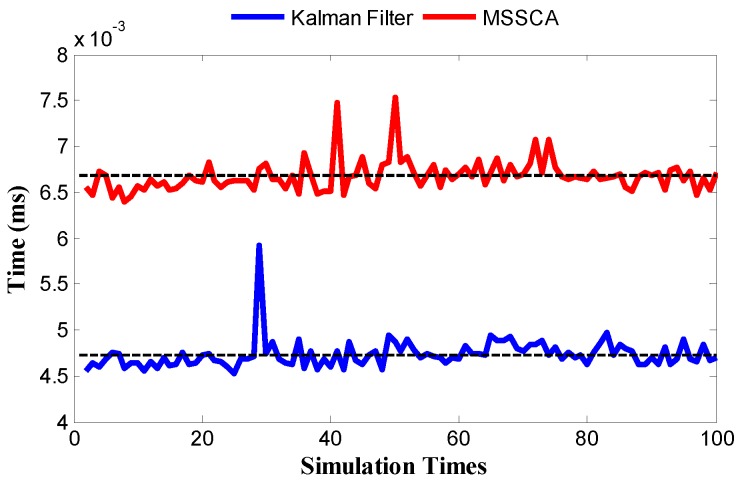
Execution time comparison between the Kalman filter and the MSSCA.

For each sensor calibration, the execution time comparison between the Kalman filter and the MSSCA is shown in Figure 16. Although the Kalman filter is clearly faster than the MSSCA, it can still achieve on-line temperature estimation. This is because the average execution time of the MSSCA (about 0.0066 ms) is obviously shorter than the sampling interval (17 ms). The requirement for our temperature estimation technique to be exploited by a processor is that additional memory is needed to store the correlation coefficients among all the available sensors.

## 6. Conclusions

In this paper, the problem of accurately estimating the temperatures for noisy thermal sensors is solved. We first analyze the noise characteristics of on-chip thermal sensors based on the ring oscillator structure and utilize the polynomial fitting technique to establish the non-linear relationship between the sensor temperature and output frequency of ring oscillator. On this basis, a smoothing filter-based Kalman prediction technique is proposed to correct the temperatures of on-die sensors in real time. Besides, a multi-sensor synergistic calibration algorithm (MSSCA) is proposed to improve the simultaneous prediction accuracy of multiple sensors. To evaluate the performance of our predictions, an infrared imaging-based temperature measurement technique is also proposed to capture the thermal traces of an AMD quad-core processor. Simulation results show that the proposed calibration scheme can achieve an around 1.2 ${}^{\circ }$C reduction in RMSE, a 15.8 dB increment in SNR, and a 28.6% reduction in FAR, as compared to the original sensor readings. Our approach will assist DTM mechanisms to achieve accurate temperature estimations in response to inaccuracies caused by fabrication randomness and environmental variation.

## Figures and Tables

**Table 1:** Characteristics of the random variables.

Parameters	$W_{n}/W_{p}$ (nm)	$L_{n}/L_{p}$ (nm)	$T_{\mathrm{ox}}$ (nm)	$V_{\mathrm{DD}}$ (v)	$V_{t}$ (v)	$\mu_{n}/\mu_{p}$ m^2^/(V·s)
Mean	270	180	4.1	3	0.45	0.034
Standard deviation	5%	6%	3%	5%	4%	2%

**Table 2:** Correlation coefficients among different sensors.

Correlation	P1	P2	P3
P1	1	0.8991	0.7134
P2	0.8991	1	0.8808
P3	0.7134	0.8808	1

**Table 3:** Average prediction accuracy for different benchmarks.

Standard Deviation	Sensor ID	RMSE (${}^{\circ }C$)			SNR (dB)		
		Sensor Readings	Kalman Filter	MSSCA	Sensor Readings	Kalman Filter	MSSCA
5%	P1	0.6757	0.2006	0.1690	−3.6697	6.8931	8.3156
	P2	0.6687	0.1913	0.1612	−4.1133	6.7305	8.2271
	P3	0.6734	0.1952	0.1651	−3.6588	7.0747	8.4353
10%	P1	1.3571	0.2776	0.2309	−9.7271	4.0771	5.6569
	P2	1.3767	0.2672	0.2193	−10.2051	3.8401	5.5996
	P3	1.3527	0.2751	0.2301	−9.7174	4.1676	5.6734

**Table 4:** Average prediction accuracy increment of the MSSCA compared with the Kalman filter.

Standard Deviation	Sensor ID	RMSE (${}^{\circ }C$)	SNR (dB)
		MSSCA vs. Kalman Filter	MSSCA vs. Kalman Filter
5%	P1	−15.75%	+20.64%
	P2	−15.73%	+22.24%
	P3	−15.42%	+19.23%
10%	P1	−16.82%	+38.75%
	P2	−17.93%	+45.82%
	P3	−16.36%	+36.13%

**Table 5:** Average FAR for different benchmarks.

Standard Deviation	Sensor ID	FAR (%)		
		Sensor Readings	Kalman Filter	MSSCA
5%	P1	28.7542	9.9867	7.1483
	P2	27.3608	10.4633	7.68583
	P3	28.0150	9.8583	7.08583
10%	P1	37.8958	13.6108	9.2775
	P2	36.9525	14.2808	10.1975
	P3	37.2767	12.6075	8.8508
